# Small-Scale Spatial Variation in Population Dynamics and Fishermen Response in a Coastal Marine Fishery

**DOI:** 10.1371/journal.pone.0052837

**Published:** 2012-12-31

**Authors:** Jono R. Wilson, Matthew C. Kay, John Colgate, Roy Qi, Hunter S. Lenihan

**Affiliations:** 1 Bren School of Environmental Science and Management, University of California Santa Barbara, Santa Barbara, California, United States of America; 2 Earth Research Institute, University of California Santa Barbara, Santa Barbara, California, United States of America; 3 Commercial Fishermen of Santa Barbara, Santa Barbara, California, United States of America; 4 College of Creative Studies, University of California Santa Barbara, Santa Barbara, California, United States of America; University of California San Diego, United States of America

## Abstract

A major challenge for small-scale fisheries management is high spatial variability in the demography and life history characteristics of target species. Implementation of local management actions that can reduce overfishing and maximize yields requires quantifying ecological heterogeneity at small spatial scales and is therefore limited by available resources and data. Collaborative fisheries research (CFR) is an effective means to collect essential fishery information at local scales, and to develop the social, technical, and logistical framework for fisheries management innovation. We used a CFR approach with fishing partners to collect and analyze geographically precise demographic information for grass rockfish (*Sebastes rastrelliger*), a sedentary, nearshore species harvested in the live fish fishery on the West Coast of the USA. Data were used to estimate geographically distinct growth rates, ages, mortality, and length frequency distributions in two environmental subregions of the Santa Barbara Channel, CA, USA. Results indicated the existence of two subpopulations; one located in the relatively cold, high productivity western Channel, and another in the relatively warm, low productivity eastern Channel. We parameterized yield per recruit models, the results of which suggested nearly twice as much yield per recruit in the high productivity subregion relative to the low productivity subregion. The spatial distribution of fishing in the two environmental subregions demonstrated a similar pattern to the yield per recruit outputs with greater landings, effort, and catch per unit effort in the high productivity subregion relative to the low productivity subregion. Understanding how spatial variability in stock dynamics translates to variability in fishery yield and distribution of effort is important to developing management plans that maximize fishing opportunities and conservation benefits at local scales.

## Introduction

Biological parameters used to manage harvested marine fishes include such life history characteristics as age at maturity, size at age, and fecundity, as well as demographic rates such as growth, and mortality. For many nearshore species, these parameters can vary geographically due to spatial variability in biotic and abiotic factors such as temperature, food, habitat, and species interactions [1]–[3]. Conventional approaches to managing nearshore fisheries often ignore spatial variability in life history characteristics and demographic rates, and instead pool these attributes across broad geographic scales [4]. The failure to account for such spatial variability may present a mismatch in the spatial scale of ecological dynamics and management actions for many nearshore fisheries [5]–[7]. This mismatch in scales can lead to underutilization and localized depletions of populations [8], often contributing to stakeholder discontent and reduced ecosystem integrity. Spatial fisheries ecology is becoming a central focus of research in the fisheries sciences and has spurred a movement towards spatial fisheries management approaches. The transition to and widespread acceptance of spatial management approaches in part depends on our ability to identify variable life history and demographic rates within and between populations, and to do so in a cost-effective manner.

Collaborative fisheries research (CFR) involving fishermen and scientists can greatly improve the spatial and temporal scale at which data is collected [9], such that assessment of populations at local scales may be a viable and cost effective option [10], [11]. Collaborative research follows the principle that those affected by a decision should be included in all phases of the decision making process, thus increasing the legitimacy and probability of acceptance of management decisions [12]. Furthermore, collaborative research is a means for both scientists and fishermen to learn from one another and identify problems and solutions that each other may be unaware of [13]. With a growing awareness of the need to utilize local information to manage small-scale fisheries [2], CFR may be an effective means to facilitate this process.

Here we utilize CFR to explore spatial variability in life history and demography of the rocky reef-associated grass rockfish (*Sebastes rastrelliger*) in the northern Channel Islands and the Santa Barbara Channel in southern California, USA. Grass rockfish are solitary reef fish that inhabit waters from Yaquina Bay, Oregon to Bahia Playa Maria, central Baja California [14]. This species lives in the shallow intertidal to depths of 46 m, but primarily in water depths less than 10 m [15]. The species has limited adult movement [16], and larval dispersal averages 10 km generation^−1^ [17]. Within the nearshore live fish fishery, grass rockfish are targeted by individual fishermen operating small vessels in shallow water using set hook and line (sticks) and trap gear. Fish are kept alive aboard boats upon return to port where distributors pay premium prices for these live fish in order to deliver the freshest product to market. In 2009, 13.2 mt of grass rockfish were landed in California, making it the fifth highest catch among the 19 managed species in California’s Nearshore Fishery Management Plan [18].

Over a two-year period from 2008–2010, we worked with commercial fishermen in the local nearshore finfish fishery to collect length frequency data, and characterize length at age, reproductive maturity, weight at length, and natural mortality. We performed model-fitting exercises to test the hypothesis that grass rockfish exhibit geographic variability in life history and demographic rates across two environmentally distinct regions of the Santa Barbara Channel. Parameter estimates from the model fitting exercises were used to populate spatially explicit yield per recruit (YPR) analyses. We found that subtle changes in life history and demographic rates across small spatial scales can translate to large differences in YPR. These differences in YPR were verified in fisheries data that demonstrated higher landings, effort, and catch per unit effort (CPUE) in the region predicted to have higher YPR. YPR models were also used to evaluate whether the current minimum size limit in the fishery maximizes YPR, and whether adjustments to the minimum size limit at spatially explicit scales can increase YPR. Results from this research have important implications for development of spatial fisheries management policies and contribute to a growing literature on the ubiquity of spatial variability in nearshore marine fisheries.

## Materials and Methods

### Ethics Statement

All fish used for laboratory analyses were humanely euthanized following the protocols set forth by the Institute for Animal Care and Use Committee (protocol #732) at the University of California Santa Barbara. Permission to conduct sampling in the Channel Islands State Marine Protected Areas was granted by the California Department of Fish and Game (SC-007197). Permission to sample in the Channel Islands National Marine Sanctuary was granted by NOAA Channel Islands National Marine Sanctuary (CINMS-2009-005).

### Study Region

**Figure 1:**
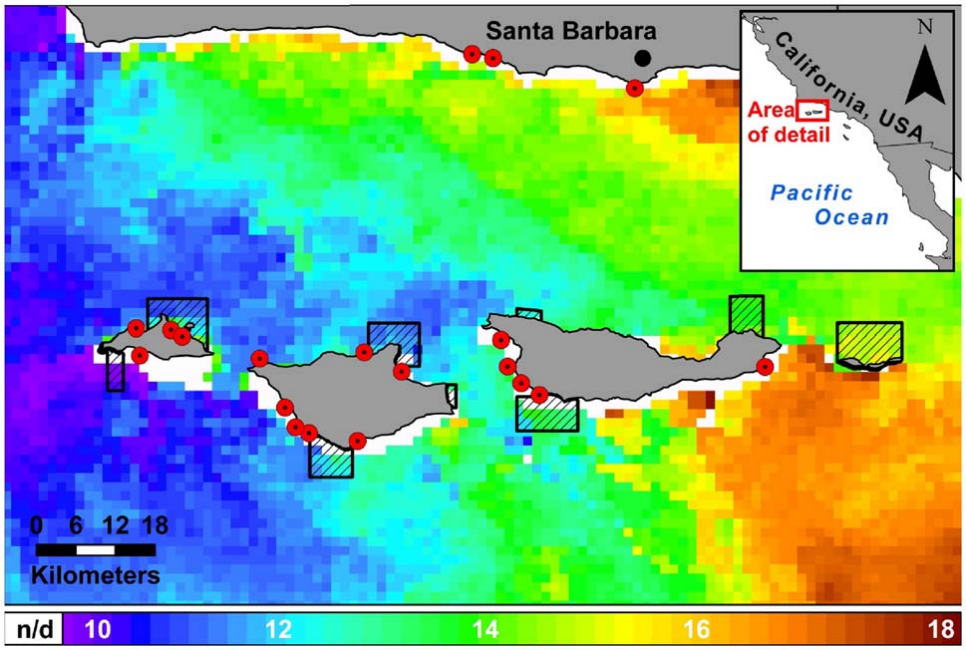
Map of the study region. Map of the Santa Barbara Channel, including the mainland coast and four northern Channel Islands (left to right: San Miguel, Santa Rosa, Santa Cruz, and Anacapa), our sampling sites (red dots), and a sea surface temperature profile averaged over a 14 day period centered on 10 June 2010 (satellite data from MODIS Aqua, downloaded from NOAA at: http://coastwatch.pfel.noaa.gov). Temperatures indicated in the ribbon legend are °C, and white cells in the map indicate areas for which no data (n/d) were available. In our study, high productivity sites were those sampled at San Miguel and Santa Rosa, whereas low productivity sites were located along Santa Cruz and the mainland coast.

The Santa Barbara Channel (SBC) ecosystem is situated in a biogeographic transition zone at the confluence of the southward flowing California Current and the northward flowing California countercurrent. In the western portion of the channel, the California current brings nutrient rich, cooler, upwelled water, while the east channel is bathed in warmer, relatively nutrient poor water [19]. Environmental variability in sea surface temperature (SST), productivity, wind stress, and wave exposure has been described over the 100 km transition zone [20]–[22]. Satellite derived estimates of SST display a strong gradient from east to west that can vary up to 8°C during certain times of the year (Figure 1). A suite of studies has characterized spatial differences in community structure, somatic growth rates, and recruitment for intertidal and kelp forest species across the channel [19], [23]–[24].

### Collaborative Research

In 2007, we developed a CFR program between scientists and commercial fishermen in the nearshore live fish fishery in the SBC. Catches in this fishery primarily occur at the northern Channel Islands and the mainland coast off Santa Barbara, CA. During a series of meetings with our commercial fishery partners, we developed a testable hypothesis to explore whether higher landings in the high productivity subregion relative to the low productivity subregion are supported by spatial differences in life history and demographic rates between the two subregions. We constructed yield per recruit (YPR) curves in each subregion using estimates of biological parameters and examined empirical patterns of fishery performance (landings, effort, and CPUE) to evaluate subregional differences in fisheries landings and predicted YPR. Furthermore, we examined whether the current minimum size limit of 30.4 cm maximizes YPR in the fishery and whether alternative options produce higher yields.

In collaboration with three commercial fishermen with a combined 90 years of fishing experience at the Channel Islands, we selected 19 sampling sites that had historically yielded high catch rates of grass rockfish, and were dominated by known grass rockfish habitats including surfgrass (*Phyllospadix spp.*), ribbon kelp (*Egregia sp.*) and/or giant kelp (*Macrocystis pyrifera*). Sampling sites were located throughout the SBC and were clustered at Santa Cruz Island, Santa Rosa Island, San Miguel Island, and the mainland coast off of Santa Barbara (Fig. 1). In addition to utilizing local fishermen knowledge and previous research on rocky reef fishes [19], we used differences in SST to categorically separate our sampling sites into two regions of the channel: a high productivity zone, consisting of San Miguel and Santa Rosa Islands (N = 11), and a low productivity zone, consisting of Santa Cruz Island and the mainland coast (N = 8). To justify this distinction, we determined the long-term average SST from a 4 km×4 km block off of western Santa Cruz Island and off of San Miguel Island, California, from 1981 to 2006, using data compiled by the AVHRR Pathfinder Version 5.2 SST Project and acquired from the NOAA National Oceanographic Data Center (available online: www.nodc.noaa.gov/SatelliteData/pathfinder4 km; [25]). These data indicated statistically significant differences (t-test; t_598_ = −9.95, P<0.001) in mean SST between western SCI (13.91°C ±1.73) and San Miguel Island (15.27°C ±1.73). Mainland sites and Santa Cruz Island sites were grouped together into the low productivity zone due to similar SST profiles in these two regions, as well as a wealth of research that has linked SST to life history variability [26]. We also performed likelihood ratio tests [27] of the von Bertalanffy model fits between Santa Cruz Island and the mainland coast and detected no significant difference between nested growth models fit to data separated by area and data aggregated between the two areas (see growth rate section for detailed methodology).

Scientific sampling of grass rockfish was conducted in 2008 and 2009 using commercial set hook and line gear known as “sticks”. Sticks are constructed of weighted PVC piping and are adorned with five leaders and hooks and baited with market squid (*Loligo* spp.). All gear was constructed with the aid of commercial fishermen and conformed to the specifications used by a majority of fishermen in the fishery. Each stick is dropped onto the seafloor from a small vessel, and is attached by line to an individually marked buoy. Scientific sampling consisted of setting approximately 30 sticks at a given site, allowing the sticks to soak for approximately one hour, and subsequently pulling the gear and recording location specific data described below. All scientific sampling was conducted by the lead author onboard commercial fishing vessels.

### Length Frequency Analyses

To evaluate population level consequences of geographically variable demographic rates, total lengths of individual fish were sampled to the nearest mm from each of the 19 sites across the SBC. Each fish was geographically referenced to the nearest site through communication with fishermen. All port-sampled fish were caught using similar gear and hook sizes in the same depth distribution as the scientific sampling. For all analyses of length frequency data, fish greater than the minimum size limit from both the scientific and port sampling data were combined. Collaboration with commercial fishermen occurred in two capacities, onboard scientific sampling, and portside sampling of landed catch. This collaborative sampling allowed us to generate a large sample size of individual length measurements greater than 30.4 cm (N = 3495) with which to perform our analyses.

Length frequency histograms were generated and combined for all sites within the high and low productivity subregions to test for spatial differences in size structure between the two subregions. Differences in size structure were explored using Kolmogorov-Smirnov tests (KS tests) and an analysis of variance (ANOVA) test, with region as a fixed factor and a mean of fish lengths at each of the sites as the dependent variable (N = 19). Data conformed to the assumptions of homogeneity of variance and independence among sample sites.

### Growth Rates

To determine growth rates of grass rockfish, 107 individuals were sacrificed from the low productivity subregion as well as 62 individuals from the high productivity subregion. In addition, we collected two settlement-stage, pelagic juveniles in the low productivity subregion from a nearshore recruitment sampling program conducted by the Partnership for Interdisciplinary Study of the Coastal Ocean (www.piscoweb.org). We make the reasonable assumption that the ages of these two fish (66–78 days) are representative of the settlement age in the high productivity subregion as well. All samples were analyzed by Fish Aging Services PTY of Port Arlington, Australia. Sagittal otoliths were aged by two individuals by reading transverse otolith sections. Four sections, approximately 300 µm thick, were cut through the otolith centers to ensure that the primodium of the otolith was observed. Sections from each block were cleaned, rinsed in alcohol, dried and mounted on glass microscope slides (50×76 mm) under glass cover slips using resin. Each section of the otolith was inspected, and the section with the clearest increments was chosen for aging. This was usually, but not necessarily, the section closest to the primordium. All annuli counts were made without knowledge of fish size, sex and location to avoid the potential for biasing age estimates.

All otolith readings were analyzed for intra-reader and inter-reader variability using an index of average percent error (APE; [28]). A subsample of the otoliths (25%) was re-aged for this analysis. A nonparametric bootstrap sampling with replacement was conducted 5000 times of the repeat reading data set and an APE was calculated for each of the 5000 samples. APE for the original reading of the otoliths fell within the 95% and 5% confidence intervals of the bootstrapped APE values indicating non-biased readings of the ages of grass rockfish.

Maximum likelihood with a lognormal error structure was used to fit the von Bertalanffy growth curve (eq. 1) to age and length data at each region:
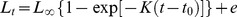
(1)where *L_t_* is the length at age t, *L_∞_* is the mean asymptotic length, *K* is the rate at which the asymptotic length is reached, and *t_0_* is the theoretical length at age zero (Table 1). We group males and females together because Love and Johnson [29] found no difference in growth rates between sexes for grass rockfish. We explored model fits to the data using several techniques to test the hypothesis of subregional differences in growth rates. We fit nested von Bertalanffy growth models to each of the subregions, and to the entire data set. We allowed for all orthogonal combinations of parameters to vary by region, totaling eight nested models. For example, model 2 allowed 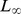
to vary by region while holding all other parameters constant between regions. An information theoretic approach was used to compare Akaike Information Criterion (AIC) values between competing nested models [27]. Lower AIC scores indicate better model fits than higher scores. The best model was chosen as the model with the lowest AIC value as well as the model with an Akaike weight of one [27]. We performed likelihood ratio tests between the null model of no regional variation in growth (model 1) and all alternative models (models 2–8). We also calculated 95% confidence intervals for the relationship between *K* and *L_∞_* [30] and visually examined the overlap to determine statistically significant differences between regions.

**Table 1:** Parameter table inlineing models, equations and parameter estimates for grass rockfish (*Sebastes rastrelliger*) in the high productivity, low productivity, and combined subregions of the Santa Barbara Channel, CA, USA.

Model		Equation	Parameters	Values		
				Combined Regions	High Prod. Region	Low Prod. Region
von Bertalanffy growth	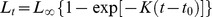		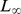	45.13	46.78	42.54
			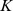	0.13	0.16	0.13
			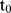	−1.32	−0.54	−1.68
Length-weight	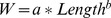		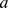	0.01	NA	NA
			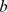	3.05	NA	NA
Length-maturity	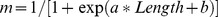		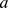	−0.49	NA	NA
			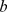	13.52	NA	NA
Fecundity	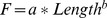		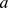	0.83	NA	NA
			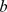	3.62	NA	NA
Naturalmortality	Average of equations 5–7(see text)		*M*	0.154	0.171	0.174

NA indicates that a single model described the fecundity and length-maturity relationships for all regions.

### Reproductive Maturity and Fecundity

Length at first maturity was estimated by visually classifying gonads as immature or mature based on criteria given in Westrheim [31], Gunderson [32] and Love and Westphal [33]. It is difficult to distinguish between immature and mature resting-stage females during the non-reproductive season [29]. Therefore, only those fish captured during the height of the reproductive season (Dec-Mar) were used for the maturity analyses. Fish were not separated by region due to low sample sizes of immature fish in the high productivity region. A logistic model was fit to the data using maximum likelihood with a lognormal error structure (Table 1).

To estimate the number of eggs at length, only those fish that were collected during the time of peak spawning were included in the fecundity analysis. This reduced our sample size to six reproductively mature females from the Santa Barbara Channel. Due to low sample sizes, we combined our data with an additional eight fish sampled by Love and Johnson in the early 1990s [29]. Fecundity was estimated following guidelines developed for the gravimetric method in Caillet et al. [34]. Low sample sizes precluded the analysis of regional variation in fecundity, so a single exponential function was fit to the data using nonlinear least squares regression that took the form *F* = *aL^b^*.

### Length-weight Relationship

Total length (mm) and weight (gm) were measured for the 169 individual grass rockfish from both regions. We fit an exponential function to the weight at length data that took the form *W* = *aL^b^*. We log transformed the data and the equation and performed three separate hypotheses tests of a nested function to explore differences in the weight at length between subregions. The nested function took on the form:
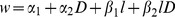
(2)where α is the log transformed *a* values, β is the log transformed *b* values, *l* is the log transformed length values, and *D* takes on a value of one in the low productivity subregion and a value of zero in the high productivity subregion. We performed a two sided *t*-test to evaluate the difference in the predicted weight at length estimates between best model fits of equation 2 to the data when one set of parameters were used to represent both populations (null model; 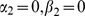), and two alternative models: 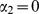 (model 1), and 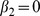 (model 2). We also performed an *F* test when α and β were allowed to be free (model 3) to explore the difference in variances of the full model.

### Yield Per Recruit, Total Landings and CPUE

Yield per recruit (YPR) is a measure of the yield of a single recruit over its lifetime [35] and takes the following form
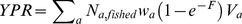
(3)where 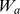 is the weight at age and *F* is the fishing mortality rate. Survivorship (*N*) to age *a* is calculated through the recursive relationship



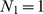


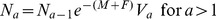
(4)


The conventional age structured approach to YPR analyses was converted into a length based model to evaluate the minimum size limit that achieves optimal YPR. To facilitate this, vulnerability at age was modeled as the probability that an individual of age *a* was above the minimum size limit (30.4 cm) according to the following logistic equation:
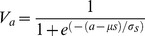
(5)where *µ_S_* is the age at 50% vulnerability to the fishing gear and *σ_S_* is the standard deviation set to 0.1.

The natural mortality rate used in this study was calculated as the average of three common life history invariant methods. The first method was a rule of thumb approach [36],
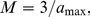
(6)where, 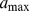 is the maximum age observed in the population. The second technique was Jensen’s method [37],
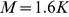
(7)where, 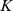 is the von Bertalanffy growth coefficient. The third technique was developed by Hoenig [38] for calculating total mortality (Z)



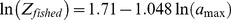
(8)This method was shown by Punt et al. [39] to be a reliable method for estimating *M* for data poor stocks. .

To determine the minimum size limit and fishing mortality rate (*F*) that would achieve optimal YPR, contour plots were generated that depict the values of YPR over a range of realistic minimum size limit adjustments between 20 and 40 cm, and a range of *F* values between zero and one, for both the high productivity region and the low productivity subregion. Yield per recruit plots were also constructed for the current minimum size limit (30.4 cm). Yield per recruit values were compared to landings (kg) of grass rockfish over the years 2000–2009 in both the high productivity subregion and the low productivity subregion. Landings data were collected by the California Department of Fish and Game through fish ticket data georeferenced to 10 nautical mile^2^ blocks. Catch per unit effort (CPUE) was calculated as the total landings by subregion divided by the total number of individual fishing trips by subregion summed across all years. CPUE was calculated in such a simplistic manner expressly to provide general insights on the relationship between CPUE, landings and productivity of the resource. Specifically, we explored the relationship between subtle changes in life history and demography, outputs of YPR analyses, and total landings, effort, and CPUE.

## Results

### Length Frequency Analyses

**Figure 2:**
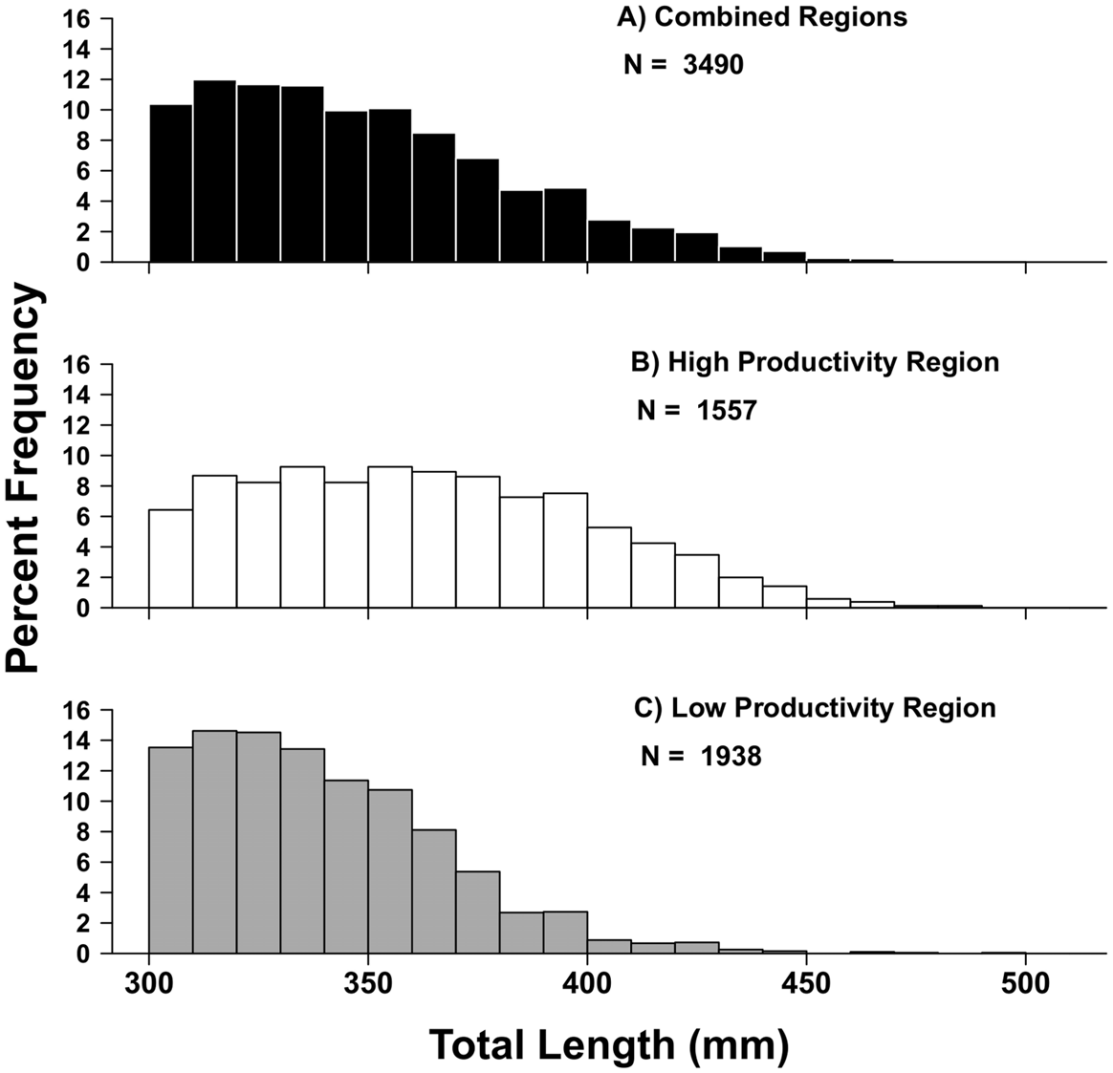
Length frequency distributions of grass rockfish (*Sebastes rastrelliger*) in the Santa Barbara Channel, CA, USA. A) All data combined across the study regions, B) length frequency distributions for the high productivity subregion, C) length frequency distributions for the low productivity subregion.

The two-year sampling period included 36 days of catch and release fishing in which 4499 sticks were set, yielding 2125 grass rockfish length samples. In addition, cooperation by several additional commercial fishermen allowed sampling of lengths to be conducted upon return to port, yielding an additional 2183 individual fish lengths from commercial landings. Results from the KS tests revealed significant differences in length frequency distributions between the high productivity subregion and the low productivity subregion (*P*<0.001; *D* = 0.287), and between each subregion and the aggregate data set in the SBC (Figure 2; *P*<0.001; *D* ≥0.243 for all combinations). The mean size of fish in the high productivity, western portion of the Channel was greater (365.62±37.55 mm) than in the low productivity subregion (342.20±27.70 mm). When data were pooled across both subregions, the mean size of fish was 352.62 (±34.48 mm). Results from the ANOVA indicated significantly greater mean sizes in the high productivity subregion than the low productivity subregion (*F*_1,17_ = 24.01, *df* = 18, *SS = *5741, *P*<0.001).

### Growth Rates

**Figure 3:**
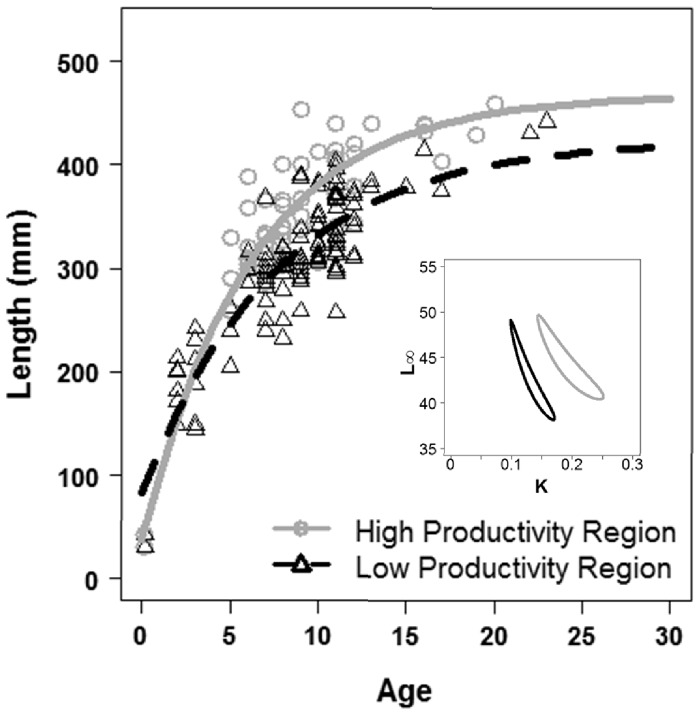
von Bertalanffy growth curves for grass rockfish (*Sebastes rastrelliger*) in the Santa Barbara Channel, CA, USA. Solid line represents the fit to data from the high productivity subregion (circles) and the dashed line represents the fit to data from the low productivity subregion (triangles) Inset depicts 95% confidence intervals around estimates of *K* and *L*_∞_ (Kimura 1980).

Results from the growth curve/aging analyses revealed statistically significant differences between the two subregions of the SBC (Figure 3). Von Bertalanffy parameter values are presented in Table 1. Comparison of the eight nested von Bertalanffy models using AIC values (Table 2) indicated that Model 5, in which all parameters are allowed to vary between subregions is the best model fit (lowest AIC: 909.50) with an Akaike weight of one. Models 6 and 7 have AIC values close to model 5 (910.9 and 910.5, respectively), which also support the hypothesis that parameters within the von Bertalanffy growth function were different between subregions. Likelihood ratio tests showed that all alternative models (2–8) were significantly different from the null model (model 1), indicating that growth data was best described by a separate von Bertalanffy growth function in each subregion (Table 2). Ninety five percent confidence intervals around the relationship between K and L_∞_ were non-overlapping (Figure 3), further supporting subregional differences in growth rates.

**Table 2:** Results of the von Bertalanffy model fitting exercise.

Model		t_0_	K		L_∞_	Ln	AIC	D	df	p-value	AIC
Likelihood											weight
1	1 region			1 region	1 region	−484.0	975.9				0.000
2	1 region			1 region	2 regions	−452.3	912.7	63.3	1	<0.001	0.204
3	1 region			2 regions	1 region	−456.1	920.2	55.7	1	<0.001	0.005
4	1 region			2 regions	2 regions	−452.2	912.3	63.7	2	<0.001	0.243
5	2 regions			2 regions	2 regions	−450.7	909.5	66.5	3	<0.001	1.000
6	2 regions			2 regions	1 region	−451.4	910.9	65.2	2	<0.001	0.504
7	2 regions			1 region	2 regions	−451.2	910.5	65.5	2	<0.001	0.613
8	2 regions			1 region	1 region	−467.7	944.9	32.6	1	<0.001	0.000

Columns 2–4 depict the orthogonal combination of parameters; one region indicates the parameter was not allowed to be different between the high and low productivity subregions, two regions indicates the parameter was allowed to vary between subregions. Ln likelihood is the negative ln likelihood of the model fit to the data. AIC = Akaike Information Criterion. D = the test statistic of the likelihood ratio test comparing alternative models (2–8) to the null model (model 1). Df = degrees of freedom. P-value refers to the likelihood ratio tests comparing alternative models to the null.

### Reproductive Maturity and Fecundity

**Figure 4:**
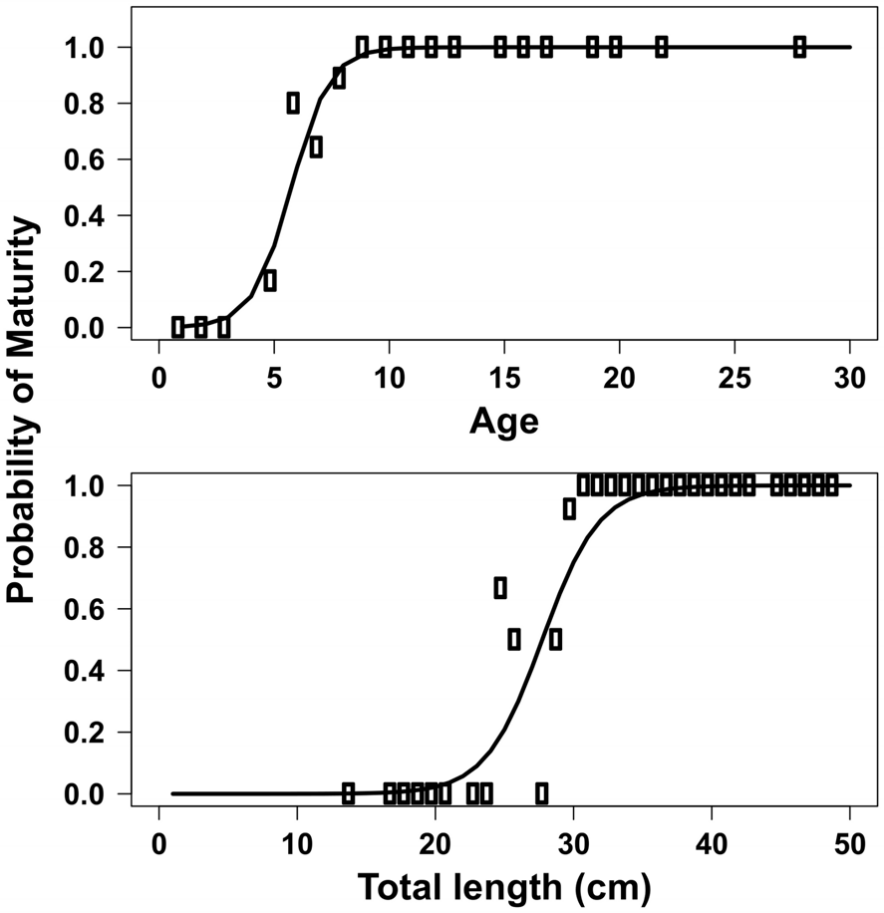
Probability of reproductive maturity for grass rockfish (*Sebastes rastrelliger*) in the Santa Barbara Channel, CA, USA. A) Probability of maturity at age, B) probability of maturity at length. Solid line represents the maximum likelihood fit to the data. .

**Figure 5:**
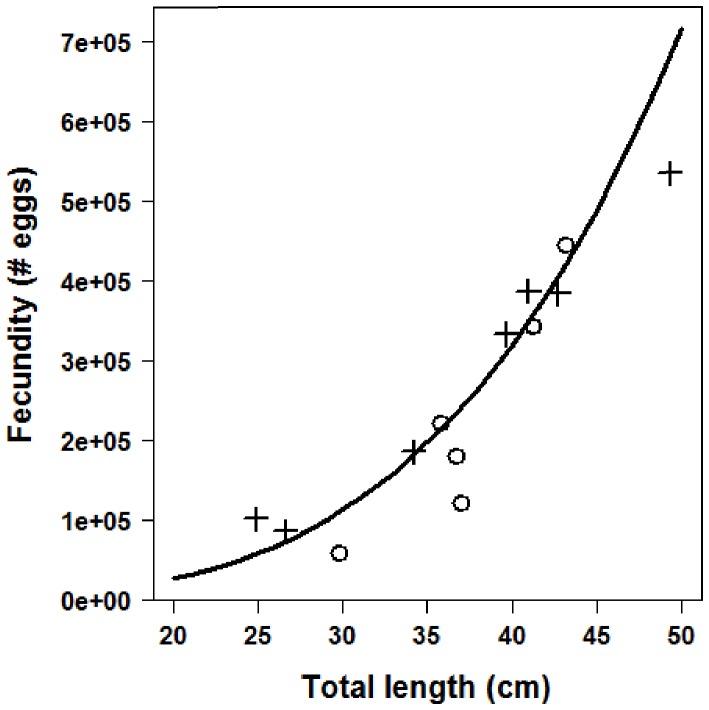
Weight at length for grass rockfish (*Sebastes rastrelliger*) in the Santa Barbara Channel, CA, USA. Solid line represents the fit to data from the high productivity subregion (circles); dashed line represents the fit to data from the low productivity subregion (triangles). Inset depicts log transformed length and weight outputs that were used for hypothesis testing.

Best-fit logistic model parameter values for length at maturity are displayed in Table 1. All grass rockfish were mature by age ten and at lengths slightly greater than the fishery’s minimum size limit of 30.4 cm (Figure 4A). Size at 50% maturity was 27.5 cm and 6 years of age. Estimates of fecundity for the six samples in this study combined with the eight samples from Love and Johnson [29] indicated a roughly six-fold increase in egg production from a 30 cm fish (100,000 eggs) to a 50 cm fish (600,000 eggs). The model fit to the data is shown in Figure 5 and parameter estimates are displayed in Table 1.

### Length-weight Relationship

**Figure 6:**
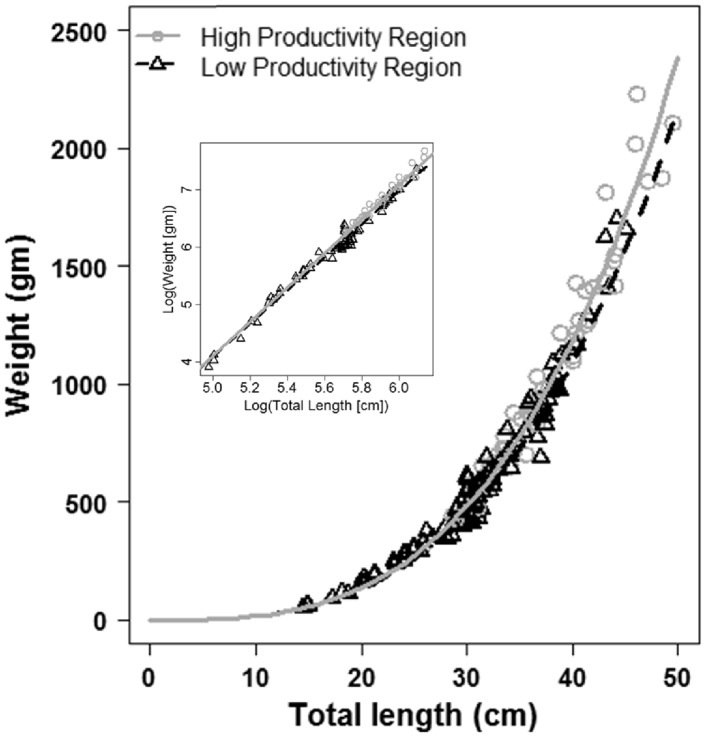
Fecundity at length for grass rockfish (*Sebastes rastrelliger*) in the Santa Barbara Channel, CA, USA. Circles represent data from the current study, and crosses represent data from Love and Johnson (1998; see text). Solid line is the best fit line.

There were no significant differences in weight at length between subregions as indicated by results from the *t*-test comparing the null model and Model 1 (*t*_210_ = 0.000, *P*>0.99), as well as the null model and Model 2 (*t*_210_ = 0.000, *P*>0.99). Results of the *F*-test further supported non-significant differences between subregions (*F_105_* = 1.003, *P*>0.987). Plots of the best-fit lines for the log transformed data and the original are depicted in Figure 6, and Table 1 presents the best-fit parameter estimates for equation 2.

### Yield Per Recruit, Total Landings, and CPUE

**Figure 7:**
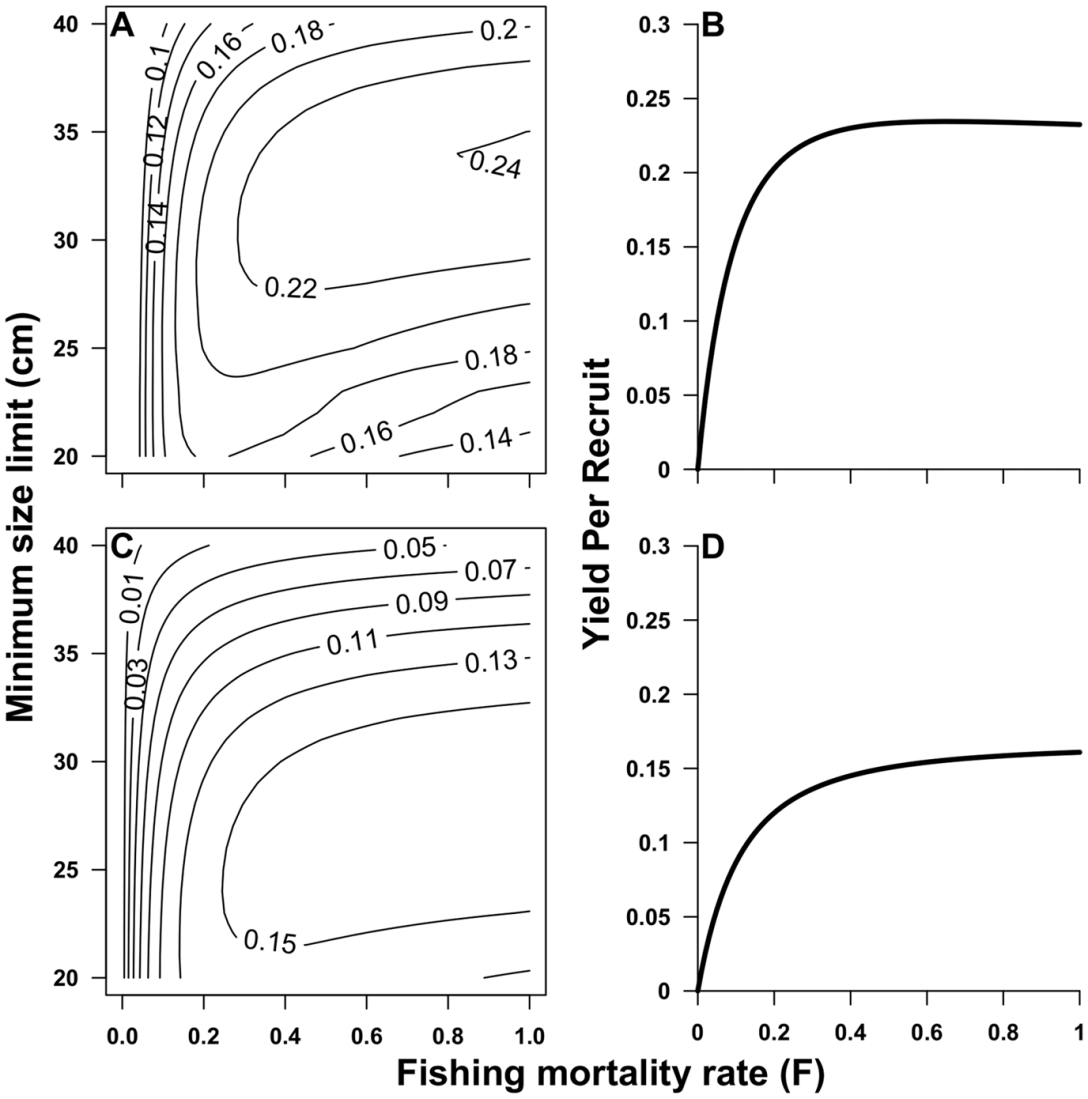
Yield per recruit for grass rockfish (*Sebastes rastrelliger*) in the Santa Barbara Channel, CA, USA. Contour plots for grass rockfish in the A) high productivity region and B) the low productivity region depict the yield per recruit at any combination of fishing mortality and minimum size limit. Figures B and D depict yield per recruit at the current minimum size limit in the high (B) and low (D) productivity subregions.

The observed age_max_ in the high productivity subregion was 28 years, while the observed age_max_ in the low productivity subregion was 23 years. Natural mortality (*M*) was marginally higher in the low productivity subregion (0.174) than the high productivity subregion (0.171). When data were aggregated across subregions, estimates of *M* were reduced to 0.154 due to presence of the oldest age classes and a low *K* value. Yield per recruit in the high productivity subregion (Fig. 7A) was roughly 1.5–2 times greater at all combinations of minimum size limit and *F*, compared to the low productivity subregion (Fig. 7C). A wide range of minimum size limits achieved high YPR at low *F* values for both subregions. In the high productivity subregion, YPR increased in association with higher *F* values, reaching highest YPR at a minimum size limit of 34 cm and *F* = 1.0. For the low productivity subregion, a similar dynamic occurred, yet YPR was generally lower and was maximized at smaller minimum size limits (22–31 cm). The existing minimum size limit of 30.4 cm performed reasonably well at maximizing YPR at lower *F* values for both subregions.

**Figure 8:**
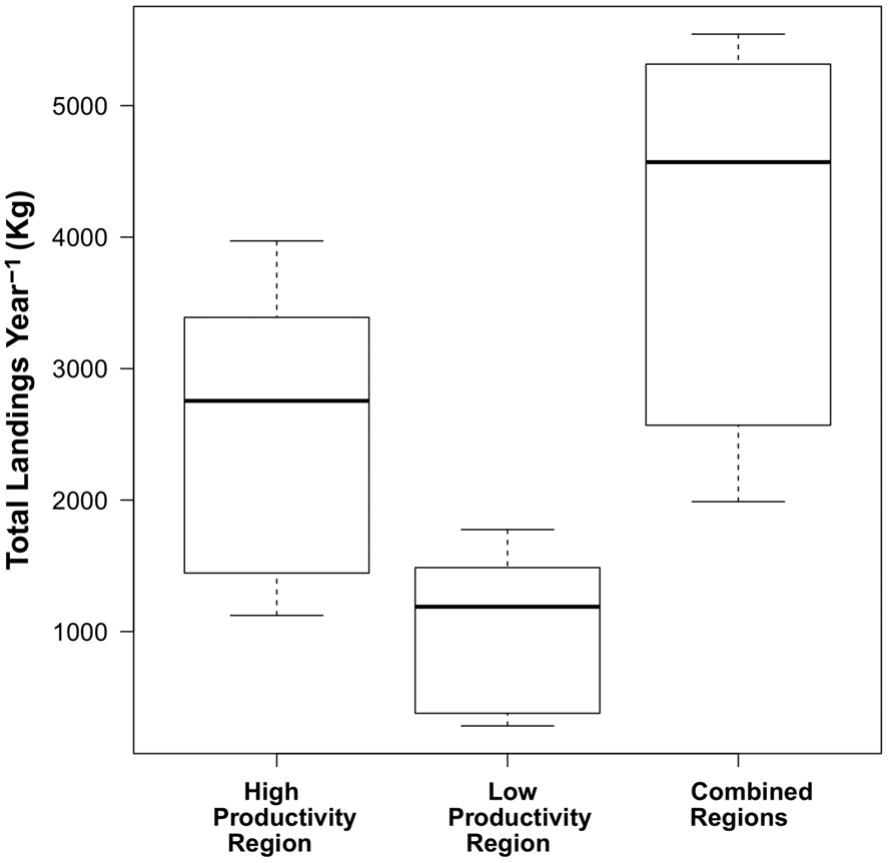
Commercial fishery landings of grass rockfish (*Sebastes rastrelliger*) in the Santa Barbara Channel, CA, USA. Box plots show the median (solid line), interquartile range (box), and 95% confisdence intervals (whiskers) of the landed grass rockfish biomass (kg.) over the years 2000–2009 for the high and low productivity subregions, and for data combined across the study regions.

Spatial variability in YPR estimates were supported by spatial differences in fishery landings, effort, and CPUE for each of the respective subregions. Total landings in the high productivity subregion ranged between 1500–3700 kg from 2000 to 2009 with a median of 3207 kg and a total of 35,712 kg. In the low productivity subregion, total landings ranged between 200–1500 kg with a median of 1660 kg (Figure 8) and a total of 18,408 kg. Roughly twice as much biomass was harvested in the high productivity subregion during the period observed in this study compared with the low productivity subregion, thus supporting the hypothesis that differences in life history and demographic variation match the rather large variation in landed biomass. Analysis of commercial fish landings data indicate that in the years 2000–2009, 892 individual trips were taken in the high productivity subregion while only 586 were taken in the low productivity subregion. CPUE in the high productivity subregion (40.04) was greater than the CPUE in the low productivity subregion (31.41), indicating that subregional variation in life history and demography also matched subregion specific effort distribution and CPUE.

## Discussion

We found that heterogeneity in fish population dynamics occurred at a small spatial scale and that fishermen effort and catch aligns with these differences in biological productivity. We found that fish in the high productivity subregion of the western Santa Barbara Channel (SBC) achieved larger asymptotic size, higher growth rates, and slightly lower natural mortality than fish in the low productivity subregion of the SBC. Population size structure and maximum age were also greatest in the high productivity zone, with the oldest age class observed being five years older than in the low productivity zone. Spatial differences in life history and demography between these two subregions translated to nearly twice as much yield per recruit (YPR) in the high productivity area. Differences in YPR were corroborated by fishery data suggesting nearly twice the fishery landings, effort, and CPUE in the high productivity subregion. Understanding spatial variability in biological productivity and fishery landings is important for the development of spatial fisheries management policies, such as the siting of marine reserves, allocation of quota in spatial catch share systems (e.g territiorial use rights in fisheries), and application of site specific catch and effort restrictions.

Higher relative YPR in the high productivity subregion resulted from larger asymptotic sizes, lower natural mortality, and older ages. Our models predicted that higher overall YPR could be achieved by raising the minimum size limit in the high productivity subregion and lowering the size limit in the low productivity subregion. However, lowering of the minimum size limit in the low productivity region may not be the best strategy for meeting the dual objectives of conservation and maximization of yields. When setting minimum size limits, it is important to consider the size at which fish become reproductively mature. YPR models do not account for the potential of high fishing mortality to reduce egg production and negatively impact recruitment. To minimize the risk of recruitment overfishing, minimum size limits should be set such that an individual can reproduce at least once before being vulnerable to harvest [40]. It would be imprudent to support a lowering of the minimum size limit in the low productivity subregion without additional analysis of the length at reproductive maturity in this subregion. The existing minimum size limit of 30.4 cm achieves high yields across the subregions and allows for several length classes to spawn before being vulnerable to the fishery, thereby minimizing the risk of recruitment overfishing [41]. Furthermore, the cost of enforcement and policy changes should be weighed against the benefits of maximizing yields in any strategy that includes fine spatial scale adjustments to minimum size limits.

Geographic variability in life history and demography for grass rockfish are probably generated from a combination of variable environmental conditions and legacy effects of historical fishing pressure. Environmental factors have been shown to influence demographic rates for a number of species. Choat and Robertson [42] described a negative relationship between sea surface temperature and maximum age for several scarids and acanthurids in the tropics. Ruttenberg et al. [1] showed that extreme spatial variability in demographic rates of *S. beebei* over a 150 km spatial range in the Galapagos Islands was largely attributed to regional variation in productivity, food availability, and water temperature. Recent work by Hamilton et al. [19] showed that in the SBC, the densities of several ecologically similar rocky reef fishes varied several fold between the high productivity zone and the low productivity zone identified in this paper. Caselle et al. [3] demonstrated spatial variation in life history and demographic characteristics across southern California for California sheephead (*Semicossyphus pulcher*), another nearshore rocky reef species that shares similar habitat and environmental conditions to grass rockfish. Hamilton et al. [43] studied California sheephead at specific sites within the high and low productivity subregions identified here and found higher von Bertalanffy *K* values at sites in the high productivity subregion as well as higher survivorship. Hamilton et al [43] attributed spatial variability in life history and demography to regional variation in environmental conditions. Grass rockfish inhabit similar kelp forest environments as California sheephead throughout the SBC and it is reasonable to assume that environmental conditions are an important driver of the regional variation observed in this study.

An alternative mechanism generating the regional variation in life history and demography that we observed is the legacy effect of historical fishing pressure (i.e., the ghosts of fishing past). The low productivity zone is closer to the ports of Santa Barbara, Ventura, and Channel Islands harbor in the east and may have received stronger historical fishing mortality during the development of the fishery in the 1980’s and 1990’s. Mechanistic links between fishing and changes in life history characteristics can be complicated by compensatory responses and changes in environmental regimes (see [44]), and unfortunately, no spatially explicit landings data from the early years of the fishery exist to test whether serial depletion occurred in this fishery [5]. The legacy effects of fishing may be responsible for the fewer older and larger individuals observed in the low productivity subregion driving down the mean asymptotic size of fish. A controlled experiment in which fishing mortality is removed from populations may help to differentiate the effects of fishing mortality from environmental pressures. In 2003, the Channel Islands State Marine Reserves were implemented in the study region and this network of ten no-take reserves may soon provide unfished estimates of life history and demographic rates.

We have demonstrated how small changes in population dynamics relate to large changes in fisheries metrics that can be used to inform sustainable management at local scales. Understanding the spatial dynamics of biological productivity can allow managers to appropriately set catch and effort regulations to ensure maximization of local yields in high productivity regions while safeguarding less productive populations from depletion. As more nearshore fisheries transition to local, community-based management, the need to gather information at small spatial scales becomes increasingly important. Our collaborative fisheries research study was a successful example of how to incorporate local fishermen knowledge in the experimental design, identification of sampling sites, and data collection process to test predictions of spatial population dynamics in commercial fisheries. Integration of fishermen into all stages of the management process is a critical step towards achieving sustainable coastal fisheries, as well as achieving cost effective and efficient monitoring and data collection policies. In conclusion, quantifying spatial heterogeneity in life history and demographic rates and how fishermen exploit these differences is key to designing effective local management strategies. Recognition of the ubiquity of spatial heterogeneity in fish stocks and the identification of this variability can help achieve the dual objectives of meeting local conservation objectives while ensuring high yields. Furthermore, knowledge of the spatial variability in fish population dynamics can be utilized to inform spatially explicit management strategies such as implementation of territorial use rights in fisheries (TURFs) and no-take marine reserves. Such information can be used to set spatially explicit size limits, spatially explicit effort allocation, and spatially explicit catch restrictions. The transition to local, community-based fisheries management will benefit from the integration of spatial management approaches and collaborative fisheries research such as the work presented here.
